# 

**Published:** 1852-02

**Authors:** 


### CONTENTS

OF NO. II.

### ORIGINAL COMMUNICATIONS.

ESSAYS AND CASES.

Art. I.—A Case of Umbilical Hernia. By M. Shepherd, M. D., of Payson, Ills. - - - - - - 93

Art. II.—A Case of Chlorosis, accompanied by Obstruction of the Bowels, which continued thirty-seven days. By Dr. P. S. Shields, of New Albany, Ia. - - - 96

Art. III.—History of the late J. Kearney Rodgers, M. D. By Alexander E. Hosack, M. D. of New York., - 101

REVIEWS.

Art. IV.—The Geological Observer. By Sir Henry T. De La Beche, C. B., F. R. S., &c. Director of the General Geological Survey of the United Kingdom. - - 117

Art. V.—Minutes of the Ohio State Medical Society, held in the city of Columbus, June, 1851, with Addresses and Essays. - - - - - - - - - - - 128

Art. VI.—Eighth Report to the Legislature of Massachusetts, relating to the registry and returns of births, marriages and deaths, in the Commonwealth. By Amasa Walker, Secretary of the Commonwealth. - - - - - 130

Art. VII.—Lecture, Introductory to the Course on the Institutes of Medicine in the University of Pennsylvania. By Samuel Jackson, M. D. - - - - - - - 138

Art. VIII.—Hints to the People upon the Profession of Medicine. By William Maxwell Wood, M. D., Surgeon U. S. Navy: author of "Sketches of South America," "Polynesia," etc. - - - - - - - - - 140

SELECTIONS FROM FOREIGN AND HOME JOURNALS.

Hæmoptysis and Tubercle, - - - - - - - - 141
Lying-in Hospital at Vienna. - - - - - - - - 149
Premonitory Signs of Severe Cerebral Disease, - - - 150
Discovery of the Male Acarus Scabiei, - - - - 156
Therapeutic Uses of Creasote, - - - - - - - 157
Disease in the Urinary Organs; Stone in the Kidney, - - 158
Cases of Acute Rheumatism cured by Lemon-Juice, - - 159
Vicarious Menstruation of a Novel Kind, - - - - - 163
A New Quackery, - - - - - - - - - - 164
Polar Cold—Probable Fate of Sir John Franklin, - - - 165
Ravages of Scurvy in Russia, - - - - - - - 172
On the Use of Opium in certain forms of Nervous Irritability and Coma, which frequently attend Typhus Fevers, - - 173
Treatment of Hemorrhoids, - - - - - - - 175
Death from Inhalation of Chloroform, - - - - - 176
Prophylaxis of Syphilis, - - - - - - - - 176

ORIGINAL INTELLIGENCE.

The Intense Cold of January, - - - - - - - 177
Case of Dr. J. K. Rodgers, - - - - - - - - 179
Modern Surgery, - - - - - - - - - - 180
Medical Attendance on Families by the Year, - - - 182
Transactions of the American Medical Association, - - 183
Modus Operandi of Cod-liver Oil, - - - - - - 183
Health of Louisville, - - - - - - - - - 184
History of Medicine in the West, - - - - - - - 184

